# Genome-Wide Identification and Characterization of the Dehydrin Gene Family in Sesame (*Sesamum indicum*): Structural Divergence and Differential Expression Under Drought Stress

**DOI:** 10.3390/genes17090998

**Published:** 2026-08-25

**Authors:** Zhangrong Chen, Hongyan Liu, Wajid Saeed, Samavia Mubeen, Sana Basharat, Qiqi Peng, Haleema Sadia, Yun Li, Muhammad Waseem, Pingwu Liu

**Affiliations:** 1 School of Breeding and Multiplication (Sanya Institute of Breeding and Multiplication), School of Tropical Agriculture and Forestry, Hainan University, Sanya 572025, China; zhangrongchen@hainanu.edu.cn (Z.C.); wajidsaeed1055@gmail.com (W.S.); sanabasharat143@gmail.com (S.B.); 2 Oil Crops Research Institute of Chinese Academy of Agricultural Sciences, Wuhan 430062, China; liuhongyan@caas.cn; 3 Guangxi Key Laboratory of Agro-Environment and Agric Products Safety, Key Laboratory of Plant Genetics and Breeding, College of Agriculture, Guangxi University, Nanning 530004, China; samavia.mubeen@gmail.com (S.M.); manuscript02@outlook.com (Q.P.); gxuliyun@gxu.edu.cn (Y.L.); 4 Department of Botany, University of Agriculture Faisalabad Pakistan, Faisalabad 38000, Pakistan; sadilatki@gmail.com

**Keywords:** dehydrin, *Sesamum indicum*, gene family, drought stress, abiotic stress

## Abstract

**Background/Objectives:** Dehydrins (DHNs) are late embryogenesis abundant proteins that play protective roles under water-deficit conditions; however, their organization and function remain unexplored in sesame (*Sesamum indicum*), an important oilseed crop frequently cultivated in arid and semi-arid regions. This study aimed to identify and characterize the DHN gene family in sesame and evaluate the expression of its members under drought stress. **Methods:** Genome-wide identification was performed using the Dehydrin domain HMM profile, followed by phylogenetic analysis, conserved motif and gene structure characterization, synteny analysis, promoter cis-element profiling, and secondary/tertiary structure prediction. Transcriptional responses were profiled by qPCR in two sesame cultivars (drought-sensitive and drought-tolerant) under PEG-induced osmotic stress at germination and seedling stages. **Results:** Four DHN genes were identified, spanning three phylogenetic subfamilies (I–III) and three DHN subclasses: SK_n_ (*SiDHN1*/2), Y_n_K_n_ (*SiDHN3*), and Y_n_SK_n_ (*SiDHN4*). *SiDHN1* and *SiDHN2* likely arose from a segmental duplication, and a single conserved syntenic pair was found between *SiDHN4* and olive (*Olea europaea*). Secondary structure predictions uncovered contrasting structural propensities: *SiDHN1*/2 are predicted to be α-helix-rich, partially ordered proteins (41–44% predicted α-helix), whereas *SiDHN3*/4 are predicted to be predominantly intrinsically disordered (~75–80% random coil). *SiDHN3* was consistently upregulated across all conditions (1.84–9.12-fold), while *SiDHN4* exhibited strong genotype-specific induction of 9.39-fold exclusively in the drought-tolerant cultivar during germination. **Conclusions:** The sesame DHN family achieves functional breadth through structural diversification—an ordered–disordered continuum mirrored by divergent expression programming—rather than through numerical expansion. *SiDHN3* and *SiDHN4* are identified as primary candidates for drought tolerance improvement in sesame.

## 1. Introduction

Drought is one of the most concerning environmental constraints limiting crop productivity worldwide. Sesame (*Sesamum indicum* L.) is an ancient oilseed crop valued for its high-quality edible oil, rich in unsaturated fatty acids and bioactive lignans [1]. It is predominantly cultivated in tropical and subtropical regions, including Africa and Asia, where seasonal water scarcity frequently compromises yield [2]. Despite its reputation as a relatively drought-tolerant crop, sesame production remains vulnerable to prolonged or severe drought events, particularly during germination and early seedling establishment [3]. Understanding the molecular mechanisms underlying drought adaptation in sesame is therefore of agronomic and biological importance.

Dehydrins (DHNs), classified as group 2 late embryogenesis abundant (LEA) proteins, constitute a family of stress-responsive proteins that accumulate in plant tissues under water-deficit conditions, including drought, salinity, and cold [4,5]. DHNs are characterized by the presence of conserved sequence motifs, most notably the K-segment (consensus KIKEKL), which is believed to form amphipathic α-helices that stabilize macromolecular structures and prevent protein aggregation during dehydration [6]. Additional conserved motifs include the Y-segment (Y-G-x), typically located near the N-terminus, and the S-segment, a serine-rich region subject to phosphorylation that may mediate nuclear localization or signal transduction [7]. Based on the combination and arrangement of these segments, DHNs can be classified into five subclasses: Y_n_SK_n_, SK_n_, K_n_, K_n_S, and Y_n_K_n_ [8]. This structural diversity is considered the basis of functional specialization across different cell types and stress conditions. It remains contentious whether the functionality of individual DHNs is predominantly mediated by ordered structural modules or by dynamic plasticity arising from intrinsic disorder [5,6].

Genome-wide characterization of DHN gene families has been performed in a wide range of plant species, including *Arabidopsis thaliana* (10 members) [9], rice (8 members) [10], and bread wheat (55 members) [11]. Individual DHN genes have been functionally characterized in olive (*Olea europaea*), where their overexpression enhances drought and salt tolerance [12,13]. These studies have revealed that the DHN family size varies considerably across taxa and that members often exhibit distinct expression patterns in response to abiotic stresses. However, no systematic identification or functional characterization of DHN genes has been reported in sesame, leaving a gap in our understanding of how this oilseed crop deploys dehydrin-mediated protective mechanisms.

Here, we investigated whether structural diversification compensated for the small size of the sesame DHN family, specifically testing whether an intrinsic ordered, disordered division among its members yielded distinctive expression programs under drought. Accordingly, we conducted a genome-wide identification of DHN genes in sesame, characterized their phylogenetic relationships, gene structures, motif compositions, chromosomal distribution, synteny patterns, and predicted secondary and tertiary structures. We then profiled transcriptional responses under PEG-induced osmotic stress across two cultivars (drought-sensitive and drought-tolerant) and two developmental stages (germination and seedling). Our results revealed a clear structural segregation between ordered and intrinsically disordered DHN members, which aligns with their divergent, context-specific expression, supporting the view that the quality of structural diversity, rather than the quantity of gene copies, may define DHN-mediated drought adaptation in this crop.

## 2. Materials and Methods

### 2.1. Genome-Wide Identification of DHN Genes in Sesame

Genome assemblies and annotation data for sesame (*S. indicum*), *Arabidopsis thaliana*, and olive (*Olea europaea* L.) were retrieved from the EnsemblPlants database (https://plants.ensembl.org/ accessed on 15 May 2026). A hidden Markov model (HMM) of the dehydrin domain (PF00257; http://pfam.xfam.org/ accessed on 15 May 2026) [14]. DHN candidate sequences were identified using the Simple HMMSearch module of TBtools-II (v2.485) with an E-value threshold of 1 × 10^−5^ and a minimum domain coverage of 50%; hits lacking a complete PF00257 domain were discarded. In addition, to recover highly divergent DHN sequences that may escape HMM-based detection, a complementary BLASTP (version 2.11.0) search was performed against the sesame proteome using the ten *Arabidopsis thaliana* DHN protein sequences as queries [9], with an E-value cutoff of 1 × 10^−5^, and the candidates recovered by the two strategies were merged into a single set. All candidate protein sequences were submitted to the NCBI Conserved Domain Database (CDD; https://www.ncbi.nlm.nih.gov/cdd/ accessed on 15 May 2026) for domain verification [15]. Sequences containing a complete DHN-characteristic conserved domain were retained; redundant transcripts, incomplete domain sequences, and abnormally sized sequences were removed. The retained sequences were further validated by BLASTP against the NCBI non-redundant protein database; only candidates whose top hits were annotated as dehydrin, or related LEA family proteins were retained. The physicochemical properties of the identified SiDHN proteins, including amino acid number, molecular weight, theoretical isoelectric point (pI), instability index, aliphatic index, and grand average of hydropathicity (GRAVY), were computed in batch using the Protein Parameter Calculator module of TBtools.

### 2.2. Phylogenetic Analysis

The DHN protein sequences from sesame, *Arabidopsis thaliana*, *Olea europaea*, *Capsicum annuum*, and *Oryza sativa* were aligned using ClustalW with default parameters. An unrooted maximum-likelihood (ML) phylogenetic tree was constructed using MEGA7.0 [16] with the JTT + G + I + F amino acid substitution model(selected as the best-fit model by BIC), and branch support was evaluated with 1000 bootstrap replicates. The tree was visualized using the iTOL online platform (https://itol.embl.de/ 15 May 2026) [17].

### 2.3. Gene Structure and Conserved Motif Analysis

Conserved motifs in the SiDHN protein sequences were predicted using the MEME algorithm implemented in TBtools, with the maximum number of detected motifs custom-set to 20 and all other parameters set to default values [18]. The gene structure (exon/intron organization) of SiDHN members was analyzed and visualized using TBtools [19].

### 2.4. Chromosomal Distribution and Synteny Analysis

Chromosomal location information of SiDHN family members was obtained from the sesame genome assembly (EnsemblPlants). Chromosomal distribution maps were generated using TBtools. Intra-species and inter-species synteny analyses were performed using MCScanX integrated within TBtools [20]. For intra-species analysis, whole protein sequence similarity searches were conducted within the sesame genome to identify syntenic blocks containing DHN genes. For inter-species analysis, sesame DHN genes were compared with the genomes of *Arabidopsis thaliana* and olive (*Olea europaea*). Syntenic relationships were performed using the R package circlize and the Advanced Circos module of TBtools [21].

### 2.5. Cis-Regulatory Element Analysis in SiDHN Promoter Sequences

The 2000 bp upstream sequences from the transcription start site of each SiDHN gene were extracted from the sesame reference genome. Cis-acting regulatory elements were predicted using the PlantCARE database (https://bioinformatics.psb.ugent.be/webtools/plantcare/html/ 15 May 2026) [22]. The identified elements were classified into three functional categories: phytohormone-responsive, abiotic and biotic stress-responsive, and plant growth and development-related.

### 2.6. Comparative Sequence Alignment and Structural Prediction

Full-length amino acid sequences of the four SiDHN proteins were aligned using ClustalW V2.1 with default parameters. Conserved dehydrin motifs, including K-, Y-, and S-segments, were identified by sequence similarity to previously reported DHN signatures. Positional conservation across the alignment was assessed using Jalview V2.11.5.2. Secondary structures of the SiDHN proteins were predicted using the SOPMA tool (https://npsa.lyon.inserm.fr/ 15 May 2026) [23]. Three-dimensional homology models were generated using the SWISS-MODEL server (https://swissmodel.expasy.org/ 15 May 2026) with templates identified from the Alpha-Fold Protein Structure Database V2.3.2 [24,25].

### 2.7. Plant Materials and Osmotic Stress Treatments

Two sesame (*S. indicum*) cultivars, one drought-sensitive (DS) and one drought-tolerant (DT), were obtained from the germplasm collection at the School of Breeding and Multiplication, Hainan University, Sanya, China. Seeds were surface-sterilized in 5% (*v*/*v*) NaClO for 10 min and thoroughly rinsed with distilled water. Seeds were placed directly on filter paper for germination and osmotic stress treatment. At the germination stage, osmotic stress was imposed by saturating seeds with 20% (*w*/*v*) polyethylene glycol (PEG) 6000, which generates an osmotic potential of approximately −0.5 MPa [26] and is widely used as an in vitro surrogate for drought, because PEG-induced osmotic stress lowers the water potential of the germination medium in a controlled, reproducible manner and thereby elicits dehydration responses comparable to those imposed by soil water deficit [27]; the treated seeds were then incubated for 72 h under controlled conditions (16 h light/8 h dark photoperiod, 28 ± 2 °C, a light intensity of 300 μmol m^−2^ s^−1^, and a relative humidity of approximately 55–60%). Control seeds were maintained on filter paper moistened with distilled water under identical conditions. Whole seedlings from both groups were harvested after 72 h. For the seedling-stage treatment, seeds were first germinated on moist filter paper at 28 °C in darkness, then transferred to hydroponic culture in perlite substrate and grown under controlled environmental conditions with Hoagland nutrient solution. Four-week-old uniform seedlings were subjected to osmotic stress by adding 20% PEG 6000 to the hydroponic culture for 12 h. For each biological replicate (*n* = 3), roots from five randomly selected plants were pooled, immediately kept in liquid nitrogen and stored at −80 °C until further analysis.

### 2.8. Quantitative Real-Time PCR Analysis

Total RNA was extracted using TRIzol (Invitrogen, Life Technologies Corporation, Carlsbad, CA, USA) reagent and treated with DNase I (Sinaclon SinaClon BioScience Co., Tehran, Iran) to remove genomic DNA contamination. RNA quality and concentration were assessed using 1.2% agarose gel electrophoresis and a NanoDrop ND-1000 spectrophotometer (Thermo Scientific, Waltham, MA, USA) based on A260/A280 ratios. First-strand cDNA was synthesized using the TransScript First-Strand cDNA Synthesis SuperMix Kit (TransGen) following the manufacturer’s protocol. For qRT-PCR, gene-specific oligonucleotides (Supplemental Table S1) targeting each SiDHN gene were designed using Primer 3.0, and SiActin (SIN_009011), a reference gene previously validated in sesame [28], served as the internal reference. Amplification was performed on an Applied Biosystems StepOne system using SYBR Premix Ex Taq (2×) in 20 µL volumes containing 10 µL master mix, 1 µL of 1:10 diluted cDNA, 0.4 µL each primer (10 µM), and 8.2 µL nuclease-free water. The thermal profile comprised an initial 30 s denaturation at 95 °C, followed by 40 cycles of 5 s at 95 °C and 30 s at 60 °C. Each biological replicate was run in triplicate (*n* = 3), and product specificity was confirmed by melting curve analysis. Primer amplification efficiencies were validated using four-point ten-fold serial dilutions of cDNA (100–10^−3^; three technical replicates per point), yielding efficiencies of 93.8–102.4% with R^2^ ≥ 0.99 for all primer pairs, which supports the use of the 2^(−ΔΔCt) method [29]. Expression ratios were computed using the 2^(−ΔΔCt) method [29]. Differences between treatment and control groups were evaluated with Welch’s *t*-test on dCt values, and the effects of cultivar, treatment, and developmental stage, together with their interactions, were assessed by three-way factorial ANOVA on dCt values; * *p* < 0.05 and ** *p* < 0.01.

## 3. Results

### 3.1. Genome-Wide Identification and Physicochemical Properties of Sesame DHN Genes

Four DHN genes were identified in the sesame genome through hidden Markov model (HMM) searches using the dehydrin domain (PF00257) as the query (E-value ≤ 1 × 10^−5^), complemented by BLASTP searches with the ten *Arabidopsis thaliana* DHN proteins to recover highly divergent homologues (Section 2.1). The recovered candidates were then verified using the NCBI CDD database. The HMM and BLASTP strategies returned an identical set of four genes, confirming that SiDHN1–SiDHN4 constitute the complete DHN family in sesame. These genes were designated SiDHN1, SiDHN2, SiDHN3, and SiDHN4 based on their chromosomal positions. The predicted proteins ranged from 150 amino acids (SiDHN3) to 235 amino acids (SiDHN1), with corresponding molecular weights ranging from 15,763.95 Da (SiDHN3) to 26,757.49 Da (SiDHN1) (Table 1). The theoretical isoelectric points (pI) varied from acidic (SiDHN1, pI 5.07; SiDHN2, pI 5.22) to near-neutral (SiDHN3, pI 6.79) and slightly basic (SiDHN4, pI 7.99), indicating electrostatic diversity among the members of this family.

All four DHN proteins were predicted to be hydrophilic, as indicated by negative grand average of hydropathicity (GRAVY) values ranging from −1.29 (SiDHN4) to −1.548 (SiDHN2). The instability index values classified SiDHN1 (56.29), SiDHN2 (65.11), and SiDHN4 (62.23) as unstable proteins (index > 40), whereas SiDHN3 (17.12) was classified as stable. The aliphatic index values ranged from 31.40 (SiDHN3) to 43.23 (SiDHN1), implying differences in thermostability. A summary of the physicochemical properties is presented in Table 1.

### 3.2. Phylogenetic Relationships of Sesame DHN Genes with Those of Other Plant Species

**Figure 1:**
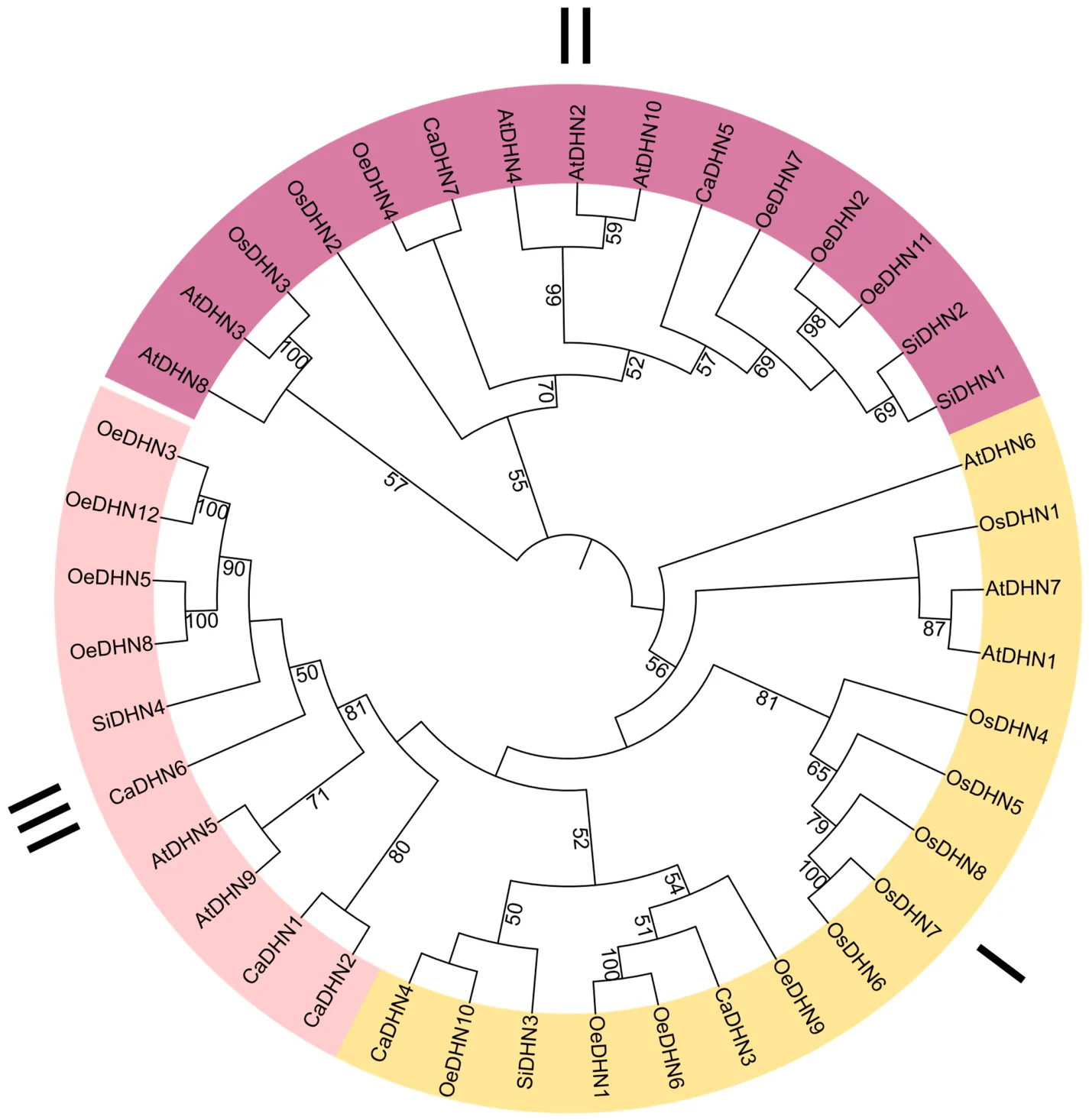
A maximum-likelihood (ML) phylogenetic tree of dehydrin (DHN) proteins identified in *Sesamum indicum* (Si), *Arabidopsis thaliana* (At), *Olea europaea* (Oe), *Capsicum annuum* (Ca), and *Oryza sativa* (Os). The proteins were clustered into three major phylogenetic groups (Subfamily I–III), indicated by different colored sectors: Subfamily I (yellow), Subfamily II (purple), and Subfamily III (pink). Protein names are labeled according to their species prefixes: Si (*S. indicum*), At (*A. thaliana*), Oe (*O. europaea*), Ca (*C. annuum*), and Os (*O. sativa*). The tree was constructed using the ML method based on full-length DHN protein sequences, and branch support was evaluated with 1000 bootstrap replicates (bootstrap support values ≥ 50% are indicated at the nodes). The colored sectors illustrate the evolutionary relationships and classification of DHN family members across the five plant species.

To investigate the evolutionary relationships among sesame DHN proteins and their counterparts in representative plant species, we constructed a maximum-likelihood (ML) phylogenetic tree using the full-length amino acid sequences of the four sesame DHNs together with 10 *Arabidopsis thaliana* [9], 12 *Olea europaea* (olive) [12,13], 7 *Capsicum annuum* (pepper) [30], and 8 *Oryza sativa* (rice) [10] DHN proteins, all identified using the PF00257 HMM profile (Section 2.1), aligned with ClustalW, and visualized with iTOL (Figure 1). The tree topology recovered three major clades, designated Subfamily I, Subfamily II, and Subfamily III, although several deep branches received low bootstrap support.

Subfamily I (yellow) contained SiDHN3 together with three Arabidopsis (AtDHN1, AtDHN6, and AtDHN7), four olive (OeDHN1, OeDHN6, OeDHN9, and OeDHN10), two pepper (CaDHN3 and CaDHN4), and six rice (OsDHN1, OsDHN4–OsDHN8) DHN proteins. Subfamily II (purple) formed the largest cluster, comprising SiDHN1 and SiDHN2 together with five Arabidopsis (AtDHN2, AtDHN3, AtDHN4, AtDHN8, and AtDHN10), four olive (OeDHN2, OeDHN4, OeDHN7, and OeDHN11), two pepper (CaDHN5 and CaDHN7), and two rice (OsDHN2 and OsDHN3) DHN proteins, which is consistent with the relatively recent segmental duplication event that gave rise to SiDHN1 and SiDHN2 (Section 3.5). Subfamily III (pink) consisted of SiDHN4 together with two Arabidopsis (AtDHN5 and AtDHN9), four olive (OeDHN3, OeDHN5, OeDHN8, and OeDHN12), and three pepper (CaDHN1, CaDHN2, and CaDHN6) DHN proteins. The presence of monocot and eudicot representatives in Subfamilies I and II, together with the eudicot-only membership of Subfamily III, confirms the ancient diversification of the DHN family in angiosperms and indicates that the tripartite subdivision is largely conserved across monocot and eudicot lineages.

### 3.3. Gene Structures and Conserved Motif Compositions

**Figure 2:**
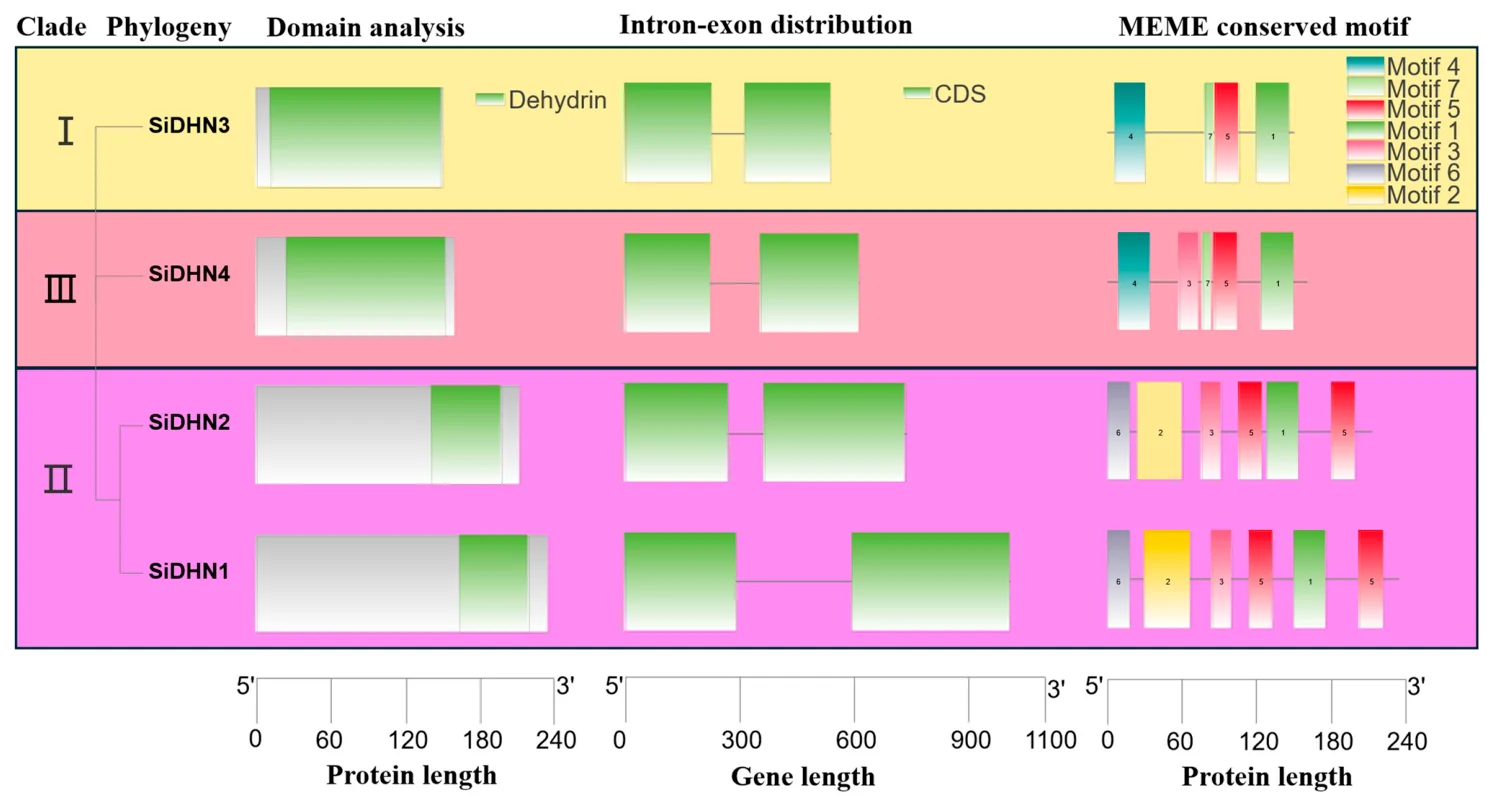
Phylogenetic relationships, gene structures, and conserved motifs of sesame *SiDHN* genes. The phylogenetic tree (left) classifies the four SiDHN proteins into three subfamilies: Subfamily I (yellow, SiDHN3), Subfamily II (purple, SiDHN1 and SiDHN2), and Subfamily III (pink, SiDHN4). Gene structures show exon–intron organization: all four genes contain two exons and one intron. Conserved motifs identified by MEME are shown as colored boxes (Motifs 1–7). SiDHN1 and SiDHN2 share an identical motif arrangement (mot6–mot2–mot3–mot5–mot1–mot5), while SiDHN3 and SiDHN4 exhibit distinct patterns.

The exon–intron organization of the four SiDHN genes was analyzed by aligning their coding sequences (CDSs) with the corresponding genomic sequences. All four SiDHN genes contained two exons and one intron, a conserved gene structure that is characteristic of plant DHN genes (Figure 2). The CDS lengths were consistent with the predicted protein sizes: *SiDHN1* (708 bp), *SiDHN2* (639 bp), *SiDHN3* (453 bp), and *SiDHN4* (483 bp). Despite the uniform exon–intron pattern, the intron lengths varied among members, contributing to differences in total gene length.

Conserved motif analysis using the MEME suite identified seven distinct motifs (mot1–mot7) among the sesame DHN proteins (Figure 2). All four proteins contained a Dehydrin domain, confirming their membership in the DHN family. SiDHN1 and SiDHN2 shared an identical motif arrangement: mot6–mot2–mot3–mot5–mot1–mot5. SiDHN3 and SiDHN4 displayed different motif compositions: SiDHN3 possessed mot4–mot7–mot5–mot1, whereas SiDHN4 contained an extended version with mot4–mot3–mot7–mot5–mot1. The shared presence of mot5 and mot1 (the K-segment-containing motif) across all members underscores the conserved core of the DHN family, while subfamily-specific motif patterns are consistent with functional divergence.

### 3.4. Chromosomal Distribution

**Figure 3:**
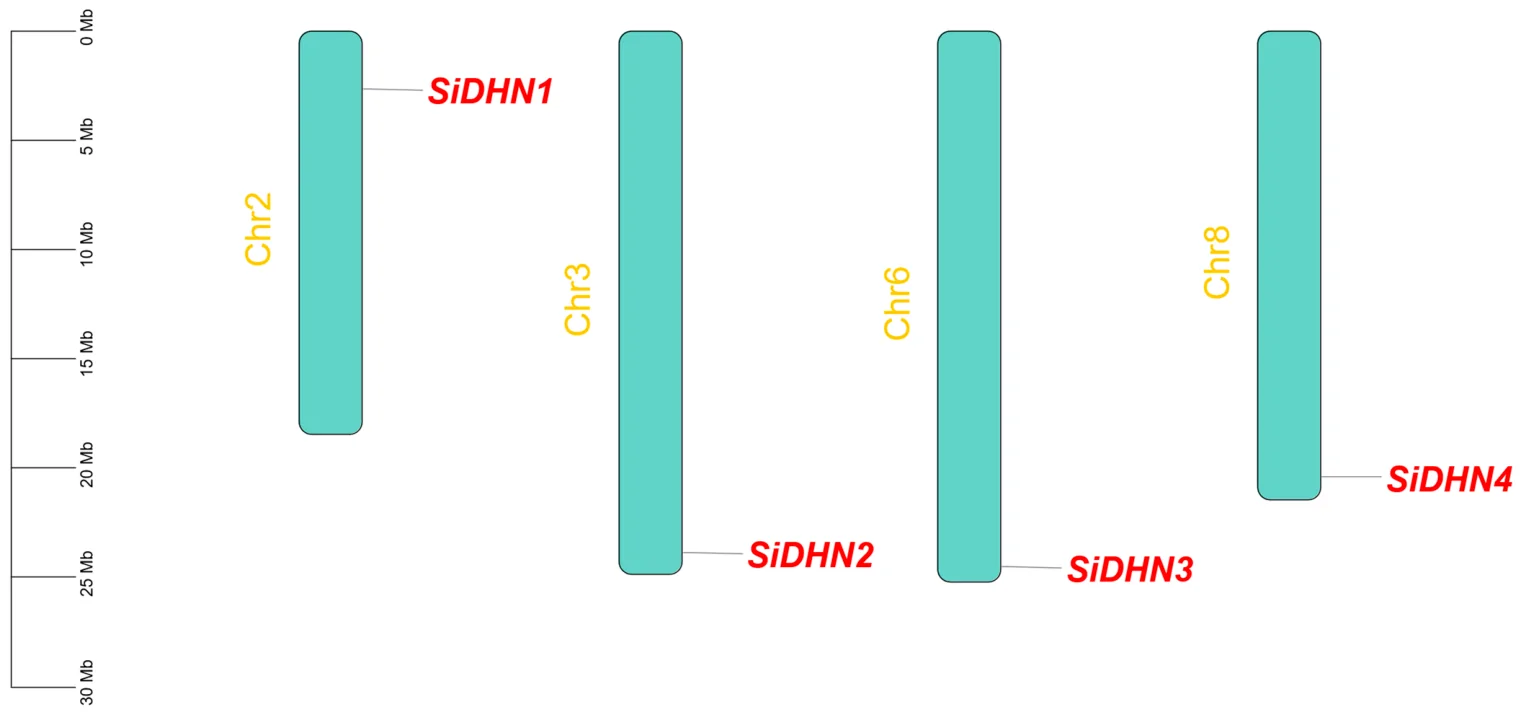
Chromosomal distribution of *SiDHN* genes in the sesame genome. The four DHN genes were distributed across four distinct chromosomes: *SiDHN1* (chromosome 2), *SiDHN2* (chromosome 3), *SiDHN3* (chromosome 6), and *SiDHN4* (chromosome 8). Chromosome numbers and gene positions are indicated in the figure legend. The figure was generated using TBtools software.

The four *SiDHN* genes were mapped onto the sesame genome. Each gene was located on a distinct chromosome: *SiDHN1* on chromosome 2, *SiDHN2* on chromosome 3, *SiDHN3* on chromosome 6, and *SiDHN4* on chromosome 8 (Figure 3), visualized using TBtools. This scattered chromosomal distribution is consistent with the pattern observed in other plant species, in which DHN genes are typically dispersed across multiple chromosomes rather than clustered in tandem arrays. The absence of tandem duplication (i.e., no two *SiDHN* genes reside on the same chromosome within a close genomic interval) is consistent with segmental or whole-genome duplication as the driving mechanism of expansion in this small family of genes.

### 3.5. Synteny Analysis: Intra-Species Segmental Duplication and Inter-Species Orthology

**Figure 4:**
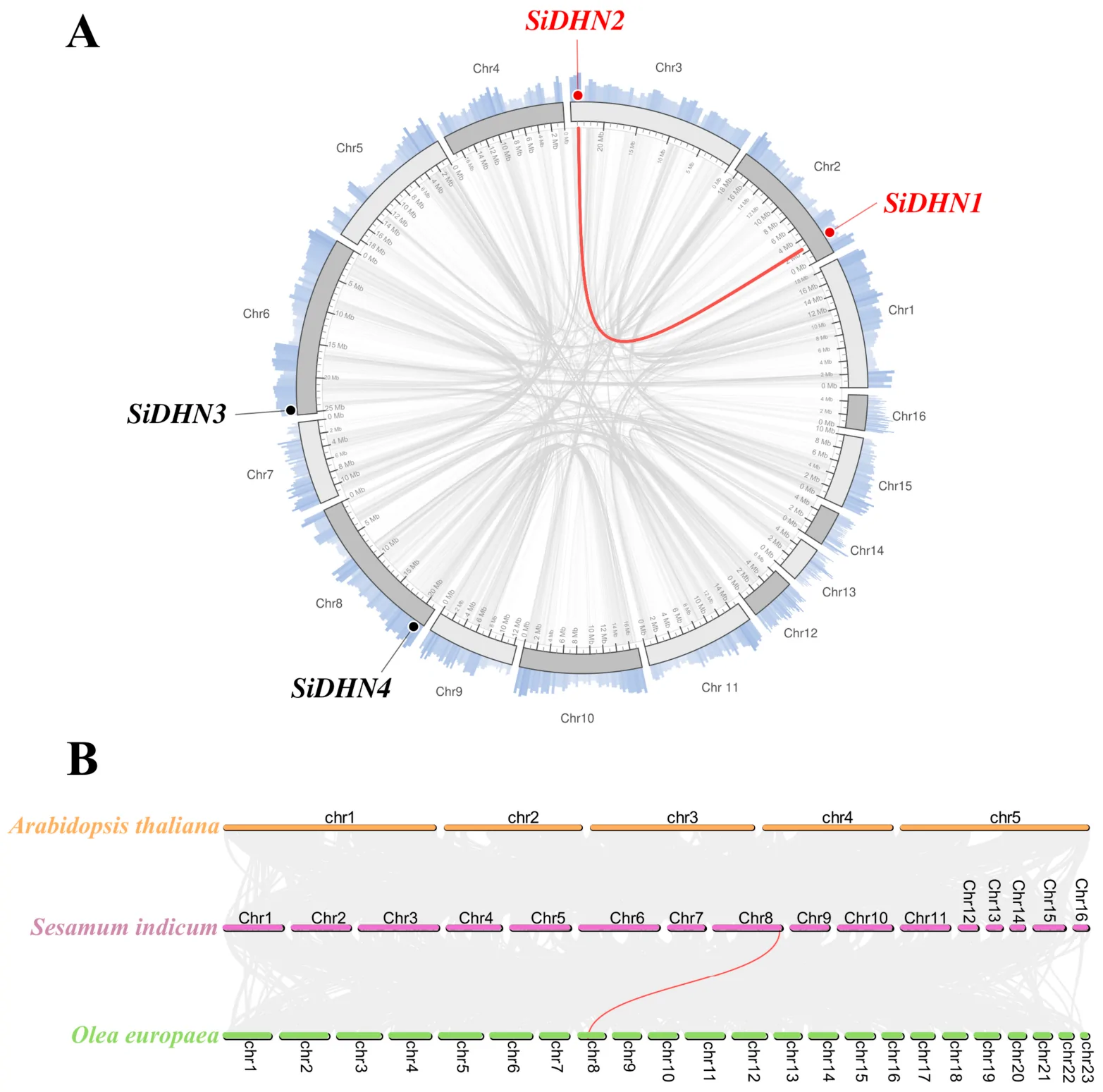
Synteny analysis of *SiDHN* genes. (**A**) Intra-species synteny of sesame *SiDHN* genes. Chromosomes are shown with alternating black and white outlines. Gray lines indicate genome-wide collinear blocks, whereas red lines highlight the duplicated *SiDHN1*–*SiDHN2* gene pair. (**B**) Inter-species synteny among *A. thaliana* (yellow), *S. indicum* (purple), and *O. europaea* (green). Red lines indicate orthologous syntenic relationships among DHN genes across species, while gray lines represent genome-wide collinear relationships.

To investigate the evolutionary mechanisms underlying the expansion of the DHN family in sesame, both intra- and inter-species synteny analyses were performed. Within the sesame genome, a significant collinear block was identified between chromosomes 2 and 3, with a syntenic score of 333.0 and an E-value of 3 × 10^−12^ (Figure 4A). This block encompassed *SiDHN1* and *SiDHN2* along with seven pairs of homologous genes, supporting the occurrence of a segmental duplication event. The inverted orientation of the collinear gene pairs within this block indicates that duplication occurred in an inverted (reverse) orientation. Together with the close phylogenetic relationship between *SiDHN1* and *SiDHN2* (Section 3.2), these results are consistent with the origin of *SiDHN1* and *SiDHN2* from a segmental duplication event; however, the timing of this duplication cannot be inferred from collinearity alone.

Inter-species synteny analysis was conducted between sesame and *Arabidopsis thaliana* and between sesame and olive (*Olea europaea*). No syntenic relationships were detected between sesame and Arabidopsis DHN genes, consistent with the substantial evolutionary divergence between the Brassicaceae and Pedaliaceae lineages. In contrast, one conserved syntenic pair was identified between sesame and olive: *SiDHN4* on sesame chromosome 8 and OE9A120446T1 on olive chromosome 8 (Figure 4B). This single syntenic pair provides preliminary evidence that the genomic context of the *SiDHN4* lineage has been maintained across the Lamiales; however, one orthologous pair is insufficient to establish long-term conservation, and additional comparative analyses will be required to support this inference.

### 3.6. Promoter Cis-Regulatory Element Analysis

**Figure 5:**
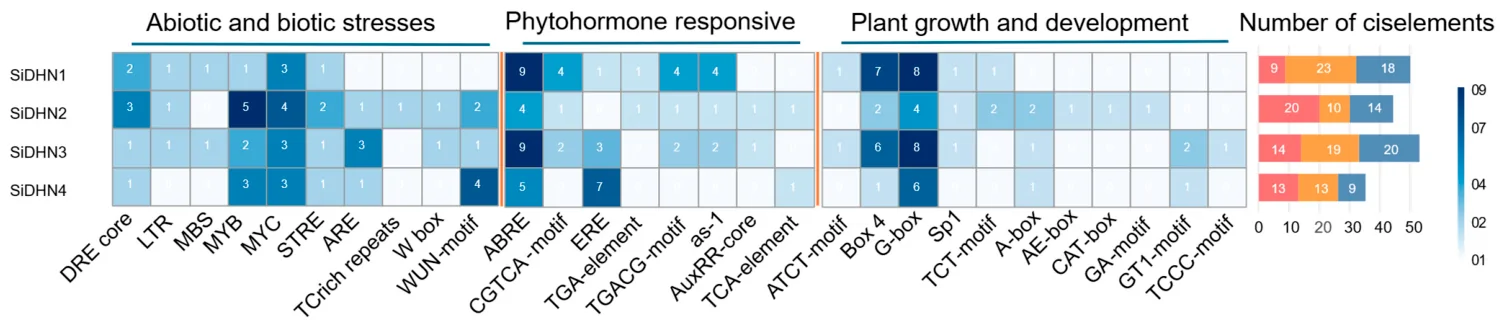
Promoter cis-regulatory elements of SiDHN genes. The 2000 bp upstream regions of SiDHN1–SiDHN4 were analyzed using PlantCARE. The identified cis-acting elements were grouped into three categories: abiotic/biotic stress-responsive, phytohormone-responsive, and growth/development-related elements. The numbers indicate the copy number of each element type within each promoter.

To gain insight into the potential regulatory mechanisms governing SiDHN gene expression, we analyzed the 2000-bp upstream promoter regions of each *SiDHN* gene using the PlantCARE database. A total of 182 cis-regulatory elements were identified, representing 29 distinct element types, which were grouped into three functional categories: hormone-responsive, abiotic/biotic stress-responsive, and growth/development-related (Figure 5).

Among the 65 hormone-responsive elements (8 types), the abscisic acid-responsive element (ABRE) was the most abundant, with 9 copies each in the promoters of *SiDHN1* and *SiDHN3*, 4 copies in *SiDHN2*, and 5 copies in *SiDHN4*, suggesting potential regulation by the ABA signalling pathway under stress conditions. CGTCA and TGACG motifs (MeJA-responsive elements) were enriched in the *SiDHN1* promoter (8 occurrences), indicating potential regulation by jasmonate signalling. The ethylene-responsive element (ERE) was most abundant in the *SiDHN4* promoter (7 occurrences).

In the abiotic/biotic stress-responsive category (56 elements, 10 types), MYB binding sites were most frequent in *SiDHN2* (5 occurrences), and MYC binding sites were also enriched in the same promoter (4 occurrences). Dehydration-responsive elements (DRE cores) were present in all four promoters, with copy numbers ranging from one to three, consistent with a potential role in stress-regulated transcription. WUN-motif (wound-responsive) elements were detected in the *SiDHN4* promoter (four copies).

Among the growth and development-related elements (61 elements, 11 types), the G-box (a light-responsive element) was prominent in *SiDHN1* and *SiDHN3* (eight copies each). Box 4, another light-responsive element, was enriched in the *SiDHN1* promoter (seven copies). The presence of diverse regulatory elements, particularly the co-occurrence of ABRE, DRE, MYB, and MYC motifs in multiple family members, suggests that SiDHN genes may be integrated into complex hormonal and stress signalling networks.

### 3.7. Sequence Alignment and Predicted Protein Structures

The secondary structures of the four sesame DHN proteins were predicted using SOPMA (Table 2). *SiDHN1* and *SiDHN2* exhibited α-helix-rich compositions, with α-helical content of 41.28% (*SiDHN1*) and 43.87% (*SiDHN2*), and random coil proportions of 58.72% and 54.72%, respectively. Beta-sheet content was negligible (0% in *SiDHN1*, 1.42% in *SiDHN2*). In marked contrast, *SiDHN3* and *SiDHN4* were dominated by random coil (80.00% and 75.00%, respectively), with α-helical content of only 15.33% and 15.62%, respectively. These computational predictions indicate that *SiDHN1* and *SiDHN2* are α-helix-rich, partially ordered proteins, whereas *SiDHN3* and *SiDHN4* are predominantly intrinsically disordered, a property commonly associated with flexible conformational sampling and molecular chaperone-like activity.

**Figure 6:**
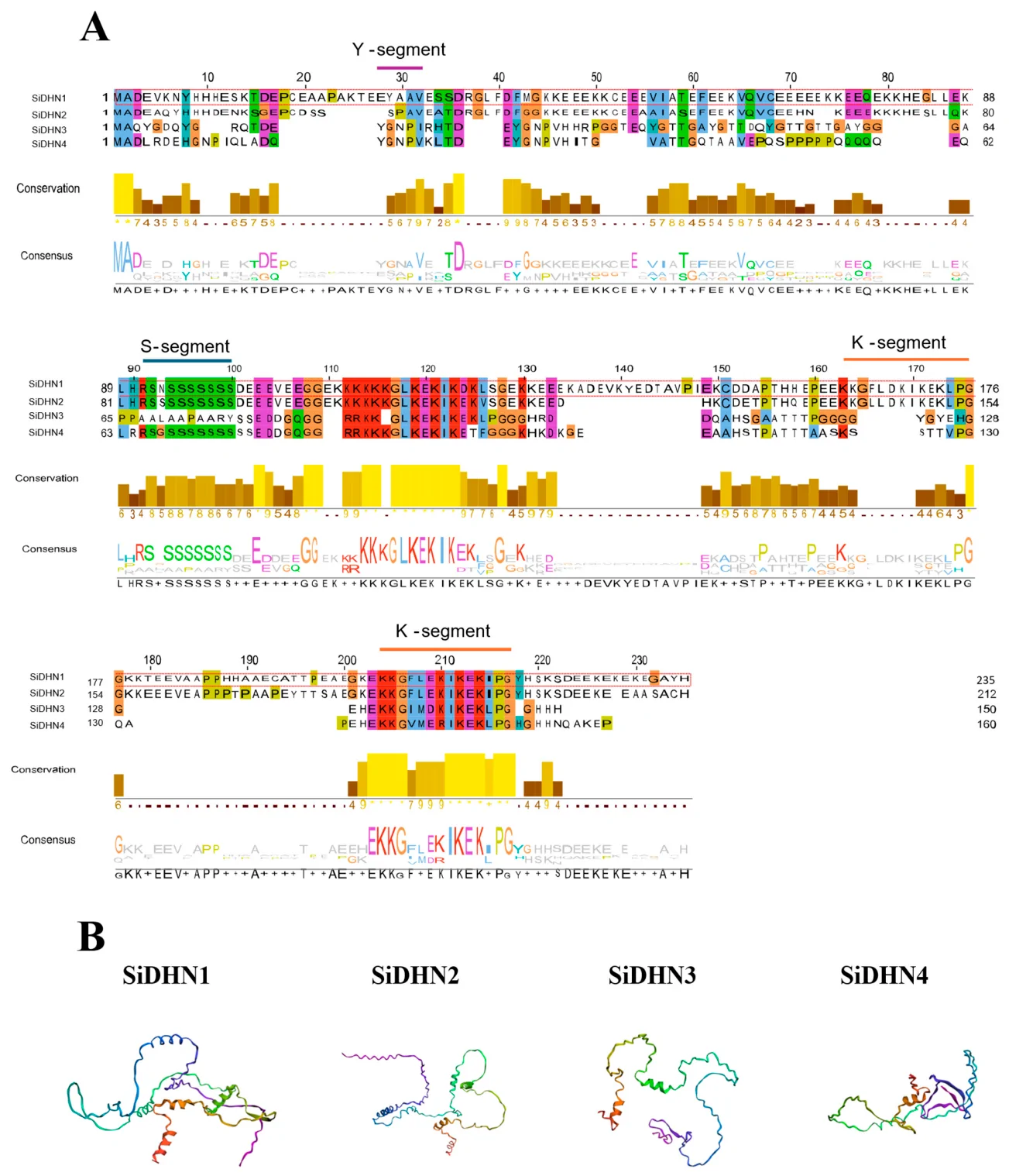
(**A**) Jalview amino acid sequence alignment of sesame SiDHN proteins showing the conserved Y-, S-, and K-segments. Y-segments are highlighted in **purple**, S-segments in **blue**, and K-segments in **orange**; **dashed boxes** delineate the respective conserved motif regions. In the alignment conservation annotation, an asterisk (*) indicates a fully conserved position, whereas a **plus sign (+)** indicates a highly conserved position in which physicochemical properties are conserved; **dashes (–)** indicate alignment gaps. SiDHN1 and SiDHN2 (SKn type) contain S- and K-segments, SiDHN3 (YnKn type) contains Y- and K-segments, and SiDHN4 (YnSKn type) contains Y-, S-, and K-segments. (**B**) Three-dimensional protein structures predicted using SWISS-MODEL. SiDHN1 and SiDHN2 exhibited relatively well-defined α-helical regions, whereas SiDHN3 and SiDHN4 showed predominantly disordered conformations.

Analysis of conserved segments revealed that all four proteins contain a K-segment, consistent with the defining feature of the dehydrin family. The canonical K-segment consensus (KIKEKL) was identified in SiDHN1 (EKKGFLDKIKEKLPGGKK, residues 162–179, and EKKGFLEKIKEKIP, residues 203–216), SiDHN2 (EKKGLLDKIKEKLPG, residues 140–154, and EKKGFLEKIKEKIP, residues 181–194), and SiDHN3 (GLKEKIKEKLPGGGHRD, residues 90–107, and EKKGIMDKIKEKLPG, residues 132–146), whereas SiDHN4 harbors a K → R variant (EKKGVMERIKEKLPG, residues 136–150; RIKEKL). The S-segment (serine-rich region) was notably longer in SiDHN1 (seven consecutive serines: SSSSSSS, residues 94–100) and SiDHN2 (nine consecutive serines: SSSSSSSSS, residues 84–92) than in SiDHN3 and SiDHN4: it is absent in SiDHN3 (only two consecutive serines, below the threshold of 3) but present in SiDHN4 (nine consecutive serines: SSSSSSSSS, residues 68–76), consistent with their respective Y_n_K_n_ and Y_n_SK_n_ subclassifications. SiDHN3 and SiDHN4 additionally possess Y-segments (Y-G-x; e.g., YGNP at residues 15–18 in SiDHN3), which are absent in SiDHN1 and SiDHN2 (Figure 6A).

Homology modelling using SWISS-MODEL, with templates sourced from the AlphaFold Protein Structure Database (AFDB), produced three-dimensional models for all four proteins, each predicted to exist as a monomer. The models for *SiDHN1* and *SiDHN2* contained higher predicted α-helical content than those for SiDHN3 and SiDHN4 (Figure 6B). In contrast, SiDHN3 and SiDHN4 were predicted to adopt extended, largely unstructured conformations, consistent with the computationally predicted disordered nature of these proteins; these conformational predictions are based solely on homology modelling and await experimental validation. Template coverage ranged from 0.987 to 1.00, and the sequence identity between the query and template sequences ranged from 93.2% to 100% (templates: A0A6I9UKK9, A0A6I9T375, A0A6I9TDZ4, and A0A6I9U3Q9 from the AlphaFold Protein Structure Database). The moderate global model quality estimates (GMQE 0.53–0.60; template pLDDT 0.53–0.60) reflect the intrinsically disordered nature of dehydrins, whose α-helices are induced upon dehydration or membrane interaction rather than being constitutively formed [5,8]; accordingly, these models should be regarded as conformational approximations, with the K-segments representing the most confidently predicted regions (template pLDDT ≥ 70 in all four proteins; pLDDT as defined in [25]).

### 3.8. Expression Profiling of SiDHN Genes Under PEG-Induced Osmotic Stress by qPCR

The transcriptional responses of the four SiDHN genes to PEG-induced osmotic stress were examined using qPCR in two sesame cultivars with contrasting drought tolerance, a drought-sensitive (DS) cultivar, and a drought-tolerant (DT) cultivar, at two developmental stages: germination (72 h of continuous PEG treatment) and seedling (roots of four-week-old plants). SiActin was used as the internal reference gene.

**Figure 7:**
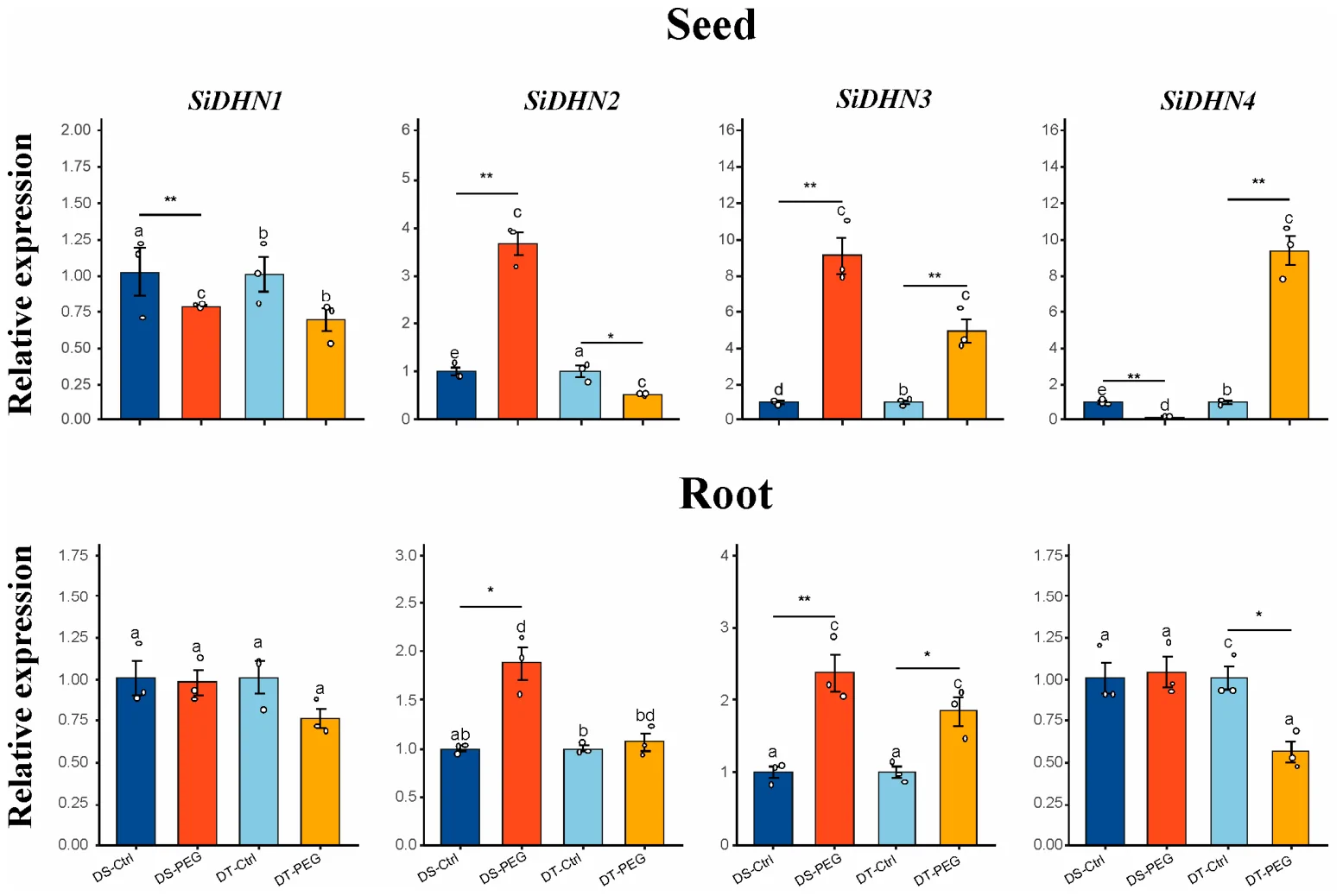
Expression profiles of SiDHN genes under PEG-induced osmotic stress. Relative expression levels of four SiDHN genes in DS and DT sesame cultivars at (**A**) germination stage (72 h treatment) and (**B**) seedling root stage (four-week-old, 12 h treatment). Fold changes were calculated using the 2^(−ΔΔCt) method, with SiActin as the reference. Bars represent the mean ± SEM; dots represent individual biological replicates (*n* = 3). Different letters above the bars indicate significant differences among the eight stage–cultivar–treatment combinations (Tukey HSD test after three-way ANOVA, *p* < 0.05); asterisks indicate significant differences between PEG-treated and control samples within each cultivar (Welch’s *t*-test: * *p* < 0.05; ** *p* < 0.01). The three-way ANOVA results are reported in Section 3.8.

To evaluate cultivar, treatment, and developmental stage as factorial factors, a three-way ANOVA was performed on dCt values for each gene. Treatment exerted highly significant main effects on SiDHN1, *SiDHN2*, and *SiDHN3* (all *p* < 0.0001), and significant cultivar × treatment interactions were detected for *SiDHN2* and *SiDHN4* (*p* < 0.0001). The three-way interaction was highly significant for *SiDHN1* (*p* < 0.0001) and *SiDHN4* (*p* < 0.0001) but not for *SiDHN3* (*p* = 0.22), consistent with the constitutive, context-independent upregulation of SiDHN3 and the strong genotype-specific induction of *SiDHN4* during germination. Individual biological replicate values are shown in Figure 7.

Germination stage. In the DS cultivar, all four SiDHN genes exhibited statistically significant expression changes under PEG-induced osmotic stress relative to control conditions (Figure 7). *SiDHN3* was strongly upregulated (9.12-fold, *p* < 0.001), and *SiDHN2 was* moderately upregulated (3.68-fold, *p* < 0.001). In contrast, *SiDHN1* and *SiDHN4* were markedly repressed (0.8-fold, *p* = 0.001 and 0.20-fold, *p* = 0.001, respectively). In the DT cultivar, a markedly different pattern emerged: *SiDHN4* showed the strongest induction (9.39-fold, *p* < 0.001), followed by SiDHN3 (4.92-fold, *p* = 0.001). *SiDHN2* was moderately downregulated (0.53-fold, *p* = 0.021), and *SiDHN1* showed a downward trend that did not reach statistical significance (*p* = 0.087).

Several notable patterns emerged from the expression data. First, *SiDHN3* was the only gene that was consistently and significantly upregulated under PEG-induced osmotic stress across both developmental stages and cultivars (*p* < 0.05 in all four comparisons), suggesting a durable and possibly central role in the sesame drought stress response. Second, the magnitude of transcriptional fold-change was substantially greater during germination (up to 9.4-fold) than at the seedling stage (maximum ~2.4-fold), indicating that the germination phase may be more responsive to DHN-mediated protective mechanisms. Third, the two cultivars displayed qualitatively distinct expression profiles: the DT cultivar showed strong induction of SiDHN4 (9.39-fold) and *SiDHN3* (4.92-fold) during germination, while the DS cultivar exhibited strong upregulation of *SiDHN3* (9.12-fold) and *SiDHN2* (3.68-fold) but marked downregulation of SiDHN1 and *SiDHN4*, suggesting that differential DHN regulation may contribute to the divergence in drought tolerance between these genotypes.

## 4. Discussion

### 4.1. The Sesame DHN Family Is Small Yet Structurally Diverse

The present study identified four DHN genes in the sesame genome, placing sesame among the species with relatively small DHN families, alongside *Arabidopsis thaliana* (10 members) [9] and *Olea europaea* (12 members identified in this study). The limited expansion of this family in sesame may reflect lineage-specific contraction or reduced reliance on DHN multiplicity given the species’ inherent drought tolerance. Despite the small family size, the four members exhibit considerable structural diversity. Phylogenetic analysis revealed that SiDHN1 and SiDHN2 are closely related and cluster together within Subfamily II, whereas SiDHN3 (Subfamily I) and SiDHN4 (Subfamily III) are more distantly related. This tripartite subdivision mirrors the organizational pattern observed across angiosperm DHN families, where ancient duplication events have generated conserved sub-lineages that are maintained across eudicot species [11,12,31]. The low bootstrap support at some deep branches of the ML tree is consistent with the short internal branches expected for DHN sequences, which are rich in intrinsically disordered regions [5,32].

The segmental duplication event that gave rise to *SiDHN1* and *SiDHN2* appears to be a relatively recent evolutionary event within the Pedaliaceae lineage, consistent with the high sequence similarity and identical motif architecture shared by these two members. The absence of tandem duplication, coupled with the presence of a single segmental duplication block, suggests that the DHN family in sesame has evolved primarily through genome-wide or large-scale duplication events rather than local gene doubling, a pattern also reported for stress-related gene families in other species [33,34].

### 4.2. Contrasting Structural Propensities Within the Sesame DHN Family

A key finding of this study was the contrasting structural propensities of *SiDHN1*/*SiDHN2* and *SiDHN3*/*SiDHN4*. The former pair is predicted to be α-helix-rich and relatively ordered (41–44% α-helix), whereas the latter pair is dominated by random coils (75–80%) and is therefore predicted to be intrinsically disordered. This structural partitioning is consistent with the broader understanding of dehydrins as a family spanning the ordered–disordered continuum [5]. *SiDHN1* and *SiDHN2*, which possess long S-segments and lack Y-segments, belong to the SK_n_ subclass and may function through more structured interactions, possibly involving membrane association or well-defined binding sites than other subclasses. In contrast, *SiDHN3* and *SiDHN4*, which contain Y-segments, belong to distinct subclasses: *SiDHN3* (lacking a canonical S-segment) is classified as Y_n_K_n_, whereas *SiDHN4* (with nine consecutive serines) is classified as Y_n_SK_n_ [35]. Their predicted disorder, a hallmark of intrinsically disordered proteins [36,37], may enable them to interact with multiple partners and exhibit chaperone-like activity under stress conditions [38,39]. However, this inference is based solely on computational structural predictions and awaits experimental validation.

The presence of a K-segment in all four members with the canonical KIKEKL consensus in *SiDHN1*/*SiDHN2*/*SiDHN3* and a K → R variant (RIKEKL) in *SiDHN4* reinforces the functional importance of this motif. The K-segment forms amphipathic α-helices that protect cellular macromolecules from dehydration-induced damage [6]. In disordered *SiDHN3* and *SiDHN4*, the K-segment may serve as a localized region of order within an otherwise flexible protein, consistent with functional studies showing that heterologous expression of dehydrins improves stress tolerance across species [40,41], potentially allowing these members to combine chaperone plasticity with targeted macromolecular stabilization.

Taken together, the sequence, phylogenetic, and structural data support a model in which the sesame DHN family achieves functional breadth not through gene expansion but through an ordered–disordered structural continuum. At one extreme, *SiDHN1* and *SiDHN2* (SK_n_ subclass, α-helix-rich, long S-segments) are predicted to adopt stable folds conducive to the formation of defined binding interfaces. At the other extreme, *SiDHN3* (Y_n_K_n_, mostly random coil, Y-segment-containing) and *SiDHN4* (Y_n_SK_n_, mostly random coil, Y- and S-segment-containing) are predicted to exhibit the hallmark properties of intrinsically disordered proteins: conformational flexibility, promiscuous interaction potential, and molecular chaperone activity. This qualitative diversification may compensate for the absence of DHN redundancy in species that inhabit inherently water-limited environments, and it predicts that the two structural classes should differ in their expression regulation.

### 4.3. Expression Divergence and Drought-Responsive Regulation

qPCR expression data revealed complex, context-dependent regulation of SiDHN genes under PEG-induced osmotic stress. The most consistent finding across all comparisons was the sustained upregulation of *SiDHN3* in both cultivars and developmental stages, suggesting that this gene plays a fundamental and non-redundant role in the drought response of sesame. The inductive response of *SiDHN3 in* the DT cultivar during germination (4.92-fold) and in both cultivars at the seedling stage (1.84–2.38-fold) suggests a potential role for this gene in the dehydration tolerance programme.

A striking observation is the 9.39-fold induction of SiDHN4 in the DT cultivar during germination, the largest fold-change measured in this study, coupled with its repression in the DS cultivar and in DT seedling roots. This stage- and genotype-specific pattern suggests that the *SiDHN4* promoter, which is enriched in ERE and WUN-motif elements, may integrate developmental and stress cues in a cultivar-dependent manner. Notably, although *SiDHN1* harbors nine ABRE copies—comparable to *SiDHN3*—its expression direction under PEG-induced osmotic stress is opposite (down-regulated vs. up-regulated). This discrepancy highlights a well-established property of ABREs: they serve as docking sites for both transcriptional activators and repressors of the bZIP family, and their net regulatory outcome depends on the wider cis-element context. The *SiDHN1* promoter lacks the ERE and WUN-motif elements present in *SiDHN3* and *SiDHN4*, which may deprive it of synergistic stress-activated transcriptional inputs; conversely, the enrichment of CGTCA/TGACG (MeJA-responsive) elements in the *SiDHN1* promoter may recruit repressive jasmonate-signalling components that override ABA-mediated activation. The abundant ABRE elements shared by *SiDHN1* and *SiDHN3* thus underscore that motif presence alone does not dictate expression polarity; rather, the combinatorial architecture of the promoter determines the integrated output [42,43].

The magnitude of expression changes during germination (up to 9.4-fold) far exceeds that observed at the seedling stage (up to ~2.4-fold). This developmental contrast may reflect the greater vulnerability of germinating seeds to water deficit: at germination, the protective seed coat has been compromised, cellular hydration is critical for radicle emergence, and the endogenous ABA/GA balance is shifted [44]. Under such conditions, rapid and strong induction of protective proteins, such as DHNs, may be essential for survival.

### 4.4. Potential for Drought Tolerance Breeding

The identification of *SiDHN3* as a constitutively drought-responsive gene and *SiDHN4* as a gene induced in a genotype-specific manner (strongly activated only in the DT cultivar during germination) offers two distinct types of candidate genes for drought tolerance improvement. The conserved and robust expression of *SiDHN3* suggests that it could serve as a universal marker for dehydration stress in sesame, while the *SiDHN4* expression pattern may represent a molecular signature of genetic variation in drought tolerance that could be exploited in marker-assisted selection or transgenic approaches [12,13]. The promoter elements identified in this study, particularly the ABRE and DRE motifs, provide a basis for further dissection of the regulatory networks controlling these genes.

### 4.5. Limitations and Future Directions

This study had several limitations. First, expression analysis was conducted in only two cultivars and two developmental stages, which constrains the generalizability of the observed patterns. A broader panel of sesame genotypes representing diverse drought tolerance levels, together with additional time points and tissues (e.g., leaves and stems), would clarify whether the expression patterns reported here are widely conserved or cultivar-specific. Second, protein structure predictions are based on computational modelling and await experimental validation by circular dichroism spectroscopy or nuclear magnetic resonance. Finally, the functional significance of individual *SiDHN* genes remains to be tested through stable genetic transformation or transient overexpression/knockdown assays in *Sesamum indicum*.

## 5. Conclusions

In this study, four DHN family members were identified, spanning three phylogenetic subfamilies and the full ordered–disordered structural continuum: from α-helix-rich, partially ordered proteins (*SiDHN1*, *SiDHN2*) to computationally predicted, predominantly intrinsically disordered polypeptides (*SiDHN3*, *SiDHN4*). This qualitative diversification, rather than numerical expansion, appears to underpin the family’s functional reach in sesame. Expression profiling supported this inference: the two structural classes exhibited distinct regulatory programs, with *SiDHN3* (disordered) showing constitutive drought-responsiveness across all conditions and *SiDHN4* (disordered) showing strong, genotype-specific upregulation exclusively in the drought-tolerant cultivar during germination. We propose that the sesame DHN family exemplifies a broader working hypothesis, namely that in species with inherently compact stress-gene repertoires, structural quality can substitute for copy-number quantity, and that the two predicted disordered members (*SiDHN3* and *SiDHN4*) are identified as the primary DHN-mediated drought-responsive candidates in this crop. Testing this hypothesis through targeted genetic manipulation, together with biophysical validation of the predicted contrasting structural propensities, will determine whether the quality-for-quantity model holds beyond sesame and represents a generalizable evolutionary strategy in dehydrin biology.

## Figures and Tables

**Table 1:** Properties of *SiDHN* gene family in *Sesamum indicum*.

Gene Name	Gene ID	Amino Acids (aa)	Molecular Weight (Da)	Theoretical (pI)	Instability Index	Aliphatic Index	GRAVY
SiDHN1	SIN_1007393	235	26,757.49	5.07	56.29	43.23	−1.533
SiDHN2	SIN_1009023	212	23,775.09	5.22	65.11	40.57	−1.548
SiDHN3	SIN_1022224	150	15,763.95	6.79	17.12	31.4	−1.293
SiDHN4	SIN_1014753	160	17,059.66	7.99	62.23	42.75	−1.29

Note: GRAVY, grand average of hydropathicity.

**Table 2:** Two-dimensional structures of SiDHN proteins in *Sesamum indicum*.

Protein Name	Gene ID	Type	Length (aa)	α-Helix (%)	β-Strand (%)	Random Coil (%)
SiDHN1	SIN_1007393	SK_n_	235 aa	41.28	0	58.72
SiDHN2	SIN_1009023	SK_n_	212 aa	43.87	1.42	54.72
SiDHN3	SIN_1022224	Y_n_K_n_	150 aa	15.33	4.67	80
SiDHN4	SIN_1014753	Y_n_SK_n_	160 aa	15.62	9.38	75

## Data Availability

The original contributions presented in this study are included in the article/Supplementary Materials. Further inquiries should be directed to the corresponding authors.

## Supplementary Materials

The following supporting information can be downloaded at: https://www.mdpi.com/article/10.3390/genes17090998/s1, Table S1: Primers used for qRT-PCR.
